# Scaling person‐centred psycho‐socioeconomic support for people living with HIV experiencing homelessness and unemployment in the Philippines: lessons learnt from the Open‐Doors Home programme

**DOI:** 10.1002/jia2.26347

**Published:** 2024-08-08

**Authors:** Rodenie A. Olete, Joseph S. Cadelina, Charmaine Faye M. Chu, Emerson A. Arriola, Inad Q. Rendon

**Affiliations:** ^1^ Gabay sa Pulang Laso Inc. (GPLI) Antipolo City Philippines; ^2^ Sustained Health Initiatives of the Philippines (SHIP) Mandaluyong City Philippines; ^3^ Department of Public Health College of Medicine National Cheng Kung University Tainan Taiwan; ^4^ Department of Sociology and Behavioral Sciences De La Salle University Metro Manila Philippines; ^5^ PHINMA – Cagayan de Oro College Cagayan de Oro City Philippines; ^6^ Innovative Inclusive Professional Team of Experts (IN+PRO) Bangkok Thailand

1

Founded in 2018, *Gabay sa Pulang Laso Inc*. (GPLI) (in English, “Guide to the Red Ribbon”) is a non‐for‐profit organization providing non‐biomedical interventions to support people living with HIV (PLHIV) in the Philippines. As a response to a nationwide survey showing significant associations between unemployment, homelessness and mental distress among PLHIV during the COVID‐19 pandemic [1], GPLI established the flagship programme, “Open Doors Home” (ODH). ODH is a temporary shelter programme with the main goal of addressing psychosocial and socioeconomic needs (also termed “psycho‐socioeconomic” or “PsySE”) among PLHIV experiencing mental distress because of homelessness, unemployment, disrupted education, domestic violence or discrimination.

The ODH programme complements biomedical interventions by providing PsySE support (i.e. shelter, nutrition, education, individualized psychosocial counselling, career guidance and livelihood trainings) as social determinants of the HIV care cascade. Guided by the person‐centred care (PCC) framework [2], individualized physical, mental, and socioeconomic needs assessments are done to ensure that the PsySE support is aligned with clients’ preferences and priorities. Clients, termed “housemates,” receive tailored PsySE support based on the individual needs assessment and individualized PCC plan. For example, if the intake interview shows that unemployment is the housemate's main concern, PsySE support will prioritize career path enhancement and referral to GPLI's network of entrepreneurs for hiring while also addressing other basic needs. ODH emphasizes empowerment, with healthcare providers serving only as facilitators to improve the housemates’ problem‐solving capacities.

Due to limited shelter capacity, a passive intake process is used where potential clients reach out via social media (Twitter/X or Facebook) or through partner organizations (e.g. HIV & AIDS Support House, Positive Action Foundation of the Philippines Inc. and other social hygiene clinics). The requirements for ODH intake are: (a) a summative case study from their medical doctor or a referral letter from the social welfare department or a community‐based organization; (b) HIV confirmatory test result; (c) a medical abstract from the last 6 months showing no concurrent opportunistic infections; and (d) copies of two valid identifications for proper coordination with their respective HIV care facilities.

Anchored on the nationwide survey [2], five fundamental building blocks of PsySE support were integrated into the ODH programme, aligning with WHO's social determinants of health:

- Housing, Basic Amenities and Environment: Provision of shelter, bedding, toiletries and other basic needs .
- Food Insecurity: Ensuring three to five meals per day.
- Access to Affordable Health Services: Healthcare services or treatment re‐initiation provided by in‐house physicians or partner HIV facilities.
- Education, Unemployment and Job Insecurity: Education support, professional career guidance and livelihood training.
- Social Inclusion and Non‐Discrimination: Supportive‐Expressive Group Therapy (SEGT).

Based on a previous study, SEGT demonstrated improved mental health after engagement within a mutually supportive group environment [3]. In ODH, SEGT was designed into four domains with 12 modules that guide housemates in expressing their emotions through focused group discussions. The modules adapted the Filipino core values of social psychology [4]. Conducted weekly over 3 months, these sessions incorporated activities like journal writing, catharsis training, role‐playing and positive reframing as different outlets of emotional expression. An on‐call psychologist is available for those experiencing severe distress (see Table 1).

**Table 1:** Philippine‐based Supportive‐Expressive Group Therapy (SEGT) module content and description as one of the PsySE elements

Modules	Session title		Description of the session
SEGT‐A: Building meaningful relationship with self	**1**	Who I truly am: Self‐awareness, self‐compassion and meaning of existence	Activities and discussion that enhance self‐awareness to understand and control thoughts, feelings and behaviours, as it is crucial for emotional intelligence, wellbeing and life satisfaction. This module aims to expand clients’ self‐knowledge, helping them navigate life by continuously knowing themselves, exploring life's purpose and implementing self‐care across different aspects.
	**2**	Positive body image, self‐perception and acceptance of physical changes	Body image encompasses an individual's subjective thoughts, beliefs and feelings about their own body. In this module, clients will be introduced to the concept of body image, aiming to cultivate a positive perception. Positive body image involves appreciating and accepting one's body.
	**3**	Discussing and developing acceptable ways of self‐expression	Self‐expression is conveyed through speech, clothing, lifestyle choices and behaviours, and communicates individuality and emotions. Understanding cultural impact is crucial for individuals. In this module, clients will explore self‐expression, linking it with prior modules on self and self‐image. The exploration will extend to understanding how living with HIV may have altered their modes of self‐expression.
SEGT‐B: Establishing long‐lasting relationships with others	**4**	Mutual understanding and support from each other in the group	A session to explore the vital role of emotional support for individuals, irrespective of HIV status. Social and cultural factors significantly influence HIV disclosure practices. This module aims to enhance interpersonal skills within the people living with HIV community for stronger emotional support and enduring external relationships.
	**5**	Emotional and social support from the family	In the Philippines, family plays a central role in decision‐making, but dynamics can hinder treatment and quality of life. This session aims to gain understanding regarding how family dynamics guides people living with HIV in seeking crucial support, positively impacting their overall wellbeing.
	**6**	Establishing meaningful collaboration and relationship with health providers	Explore the critical aspect of forming relationships with healthcare providers for individuals living with HIV. Just as family support is vital, establishing a strong bond with healthcare professionals is equally crucial. A positive connection fosters open communication, ensuring optimal HIV management and enhancing overall wellbeing, especially when accessing biomedical care.
SEGT‐C: Preparing for overwhelming emotions	**7**	Locus of control: Acknowledging what can and cannot be controlled	Delve into the vital concept of locus of control, shaping individuals’ perceptions of life's controllability. Understanding internal and external loci of control is crucial. This module aims to enlighten clients on this concept, fostering an internal locus of control to empower health decisions and enhance overall wellbeing.
	**8**	Practicing emotional catharsis in dealing with overwhelming experience	A diagnosis of HIV brings forth a range of emotions, often leading to feelings of powerlessness and isolation. This module guides clients in understanding and navigating overwhelming emotions, emphasizing the healing power of awareness and expression. It creates a secure space for clients to openly share their thoughts and feelings about their diagnosis and treatment effects.
	**9**	Identifying, processing and acknowledging trauma	Traumatic stress, a normal reaction to abnormal events, significantly impacts various aspects of life. The HIV diagnosis, medical treatments and stigma are traumatic stressors. Unresolved symptoms worsen and affect individuals negatively. This module helps clients process HIV‐related traumatic stress, fostering healthier coping skills in a safe environment.
	**10**	Gray, grief and grave: Introspective processing of the concept of death	HIV‐related bereavement, a complex phenomenon, induces psychological distress. Beyond death or chronic illness threats, an HIV diagnosis entails various losses, notably in identity. This module facilitates open discourse on death, dying and grief, aiding clients in processing experiences and improving emotional wellbeing, integrating the Kubler−Ross (1969) Model.
SEGT‐D: Developing optimistic outlook and quality of life	**11**	Identifying skills and capabilities for productivity (building a life project)	An HIV diagnosis prompts identity shifts, self‐stigma and discrimination, often leading to job loss with substantial stress, compromising the immune system. This session involves peer support programmes and people living with HIV in career decisions, fostering personal empowerment.
	**12**	Defining personal standards and developing life project	Explore personalized standards of quality of life and cultivate life projects tailored for individuals dealing with HIV, unemployment or homelessness. This session aims to empower participants in envisioning and structuring a positive and fulfilling future despite current challenges.

To monitor the mental wellbeing of the housemates during SEGT sessions, a 9‐item Patient Health Questionnaire (PHQ‐9) [5] was used to screen depression‐related symptoms, while a 7‐item Generalized Anxiety Disorder questionnaire (GAD‐7) [6] for anxiety‐related symptoms and evaluated every 2 weeks. Results from August to October 2022 from 22 housemates experiencing either homelessness or unemployment demonstrated a significant reduction in depression and anxiety after the SEGT sessions. The participants’ age ranged between 19 and 52 years old (mean = 33.3 years old, SD = 7.9). While attending the SEGT, averages in PHQ‐9 and GAD‐7 at baseline were at moderate levels (12.2 and 12.4, respectively) and significantly decreased to be at low to no risk by the last week of the SEGT session (4.8 and 4.8, respectively) [7]. Despite the limitations in the small sample size, SEGT showed potential in improving the housemates’ mental wellbeing.

ODH extends its support beyond mental wellbeing to include education, professional career guidance and livelihood training, equipping housemates with the skills needed for future opportunities. Additionally, the programme incorporates a sustainable financing element, exemplified by the establishment of a convenience store, fostering self‐sufficiency among housemates. The organization provides start‐up capital for the convenience store's grocery items, and the housemates manage the business. The net income generated each day is used as an additional food allowance in the shelter. Career path counselling is particularly emphasized during the last two SEGT sessions, where housemates are encouraged to express their future plans beyond the ODH shelter. By this stage, most housemates have initial plans to seek paid work, start an income‐generating project, or return to school. Those without concrete career directions are referred to GPLI's network of local business owners for temporary employment, such as at barbershops, convenience stores, and vegetable and meat markets, ensuring they have a source of income while planning their next steps. Among the 22 housemates who participated in the SEGT sessions from August to October 2022, 17 were linked to employment, four received educational support, and one was linked to both employment and educational support.

While the ODH programme includes a strong focus on meeting the non‐biomedical needs of clients, it also supports their biomedical needs. ODH refers housemates to in‐house physicians or partner HIV facility, ensuring that participants receive essential medical care. Adherence counselling and monitoring are crucial to ensure individuals follow their treatment plans. Throughout the ODH stay, support staff closely monitored housemates’ adherence to antiretroviral therapy (ART) through pill‐counting and verbal reminders to take their medication. An advantage of ODH is re‐engaging PLHIV with treatment, facilitated by GPLI's daily pill‐taking monitoring, weekly career tracking, and monthly/quarterly coordination with partner HIV facilities for medication refills and viral load results. A treatment re‐engagement example is a housemate from the August to October 2022 group who, after stopping HIV treatment in 2015, was promptly referred to GPLI's partner HIV facility for re‐initiation.

The success of the ODH PsySE support in enhancing the mental wellbeing and socioeconomic capacity of Filipino PLHIV underscores the need to integrate PsySE programmes into the existing HIV care cascade in the Philippines. This integration should prioritize mental health counselling and training on healthy emotional expression. Advocating for the optimal use of social welfare funding for psychosocial care aligns with the Department of Social Welfare and Development (DSWD) Administrative Order No. 4 series of 2013 [8] and No. 15 series of 2022 [9]. Strengthening collaboration with the city social welfare department is crucial for sustainable, scaled‐up PsySE support. Additionally, enhancing the ODH model involves reinforcing professional career pathway and education support programmes. Collaboration between community‐based organizations and private agencies can broaden job opportunities while reducing HIV‐related stigma and discrimination in the workplace.

The lessons learnt from the ODH programme guided the capacity‐building programme for mental health and social welfare professionals, currently implemented by the Sustained Health Initiatives of the Philippines (SHIP). As of this writing, SHIP has conducted a co‐design workshop to design the training curriculum in collaboration with Network Plus, UNAIDS, DSWD, the Department of Health, and other PLHIV‐led community‐based organizations. The goal is to widen HIV service delivery network to include mental health and social welfare professionals who can competently integrate PsySE support and other non‐biomedical needs into the HIV care cascade in the Philippines. Sustained gains after the completion of the programme warrant further investigation.

## COMPETING INTERESTS

All authors declare no competing interests.

## AUTHORS’ CONTRIBUTIONS

RAO developed the ODH framework, sought funding for implementation, designed the Supportive‐Expressive Group Therapy (SEGT) module with CMC, JSC, and IQR as co‐developers. CMC was the resident psychologist who supervised the implementation of the SEGT module and was available to do psychological interventions for clients who had mental distress. EAA was responsible for participant recruitment. JSC and CMC counterchecked the accuracy of data analysis for the GAD‐7 and PHQ‐9 monitoring. All authors provided feedback and evaluation during the manuscript development and approved the final manuscript for submission.

## FUNDING INFORMATION

The ODH programme was supported by the IAS Person‐Centred Care Programme. No funding was provided for the preparation of this manuscript.

## Data Availability

The data that support the findings of this study are available on request from the corresponding author. The data are not publicly available due to privacy or ethical restrictions.
